# Full Remission With Lurasidone in Partially Electroconvulsive Therapy-Responsive Bipolar Depression: A Case Report

**DOI:** 10.1192/j.eurpsy.2026.11186

**Published:** 2026-07-31

**Authors:** F. N. Turan Oztas, A. Erdoğan

**Affiliations:** Department of Psychiatry, Akdeniz University,Faculty of Medicine, Antalya, Türkiye

## Abstract

**Introduction:**

Bipolar disorder (BD) is a chronic mood disorder characterized by manic and depressive episodes.Several treatment strategies are available. Lurasidone is an atypical antipsychotic with an approved indication for the treatment of bipolar depression (Keramatian et al., 2023).

**Objectives:**

We present a case of bipolar depression in which the patient showed only a partial response to 20 sessions of electroconvulsive therapy(ECT), but achieved full remission after the addition of lurasidone.

**Methods:**

A 47-year-old divorced woman,living with her children,was admitted to our outpatient clinic due to suicidal ideation,depressed mood,loss of interest, impaired self-care, sleep disturbances. She had been diagnosed with BD in 2007, with her first episode being depressive, had since experienced eight episodes in total (four depressive,four manic).

**Results:**

Mental status examination revealed depressive mood, decreased psychomotor activity,active suicidal ideation.During the clinical assessment,her suicidal thoughts persisted; thus, inpatient treatment was planned. At admission, her treatment regimen included lithium 900 mg/day,lamotrigine 200 mg/day,quetiapine 100 mg/day,bupropion 450 mg/day,olanzapine 10 mg/day,mirtazapine 15 mg/day.Her Hamilton Depression Rating Scale (HAM-D) score was 36.ECT was planned. Anesthesiology consultations confirmed suitability for ECT. Lithium and lamotrigine were gradually discontinued.After 14th session of ECT her suicidal thoughts decreased,sleep problems resolved,self-care improved. She was discharged on olanzapine 20 mg/day,quetiapine 200 mg/day,mirtazapine 15 mg/day,sertraline 50 mg/day in partial remission.

At her first follow-up, depressive symptoms continued; therefore, ECT was extended to 20 sessions. Despite this, the patient remained in partial remission (HAM-D:16). Lurasidone was initiated at 40 mg/day and titrated to 80 mg/day. Following this, she achieved full remission (HAM-D:3).

**Conclusions:**

BD is a common disorder that carries a high risk of suicide, and has limited approved treatment options. In the literature, lurasidone has been reported to be effective in treatment-resistant BD cases, particularly in ultra-rapid cycling presentations with predominant depressive symptoms (Siwek & Gorostowicz, 2021). In our case, the patient did not respond to multiple antidepressants, antipsychotics, and mood stabilizers and showed only partial improvement after 20 sessions of ECT. However, the addition of lurasidone led to full remission. To our knowledge, this is the first reported case in the literature demonstrating the efficacy of lurasidone in a patient with BD partially responsive to ECT. Therefore, this report may contribute to the literature by providing an alternative therapeutic option for clinicians facing the challenge of treatment-resistant BD, particularly in cases where full remission is not achieved despite extensive ECT.

**Disclosure of Interest:**

None Declared

